# Molecular Basis of GABA Aminotransferase Inhibition in Epilepsy: Structure, Mechanisms, and Drug Development

**DOI:** 10.3390/cimb47121032

**Published:** 2025-12-11

**Authors:** Muhammad Yasir, Jongseon Choe, Jin-Hee Han, Wanjoo Chun

**Affiliations:** 1 Department of Pharmacology, Kangwon National University School of Medicine, Chuncheon 24341, Republic of Korea; yasir.khokhar1999@gmail.com; 2 Department of Microbiology and Immunology, Kangwon National University School of Medicine, Chuncheon 24341, Republic of Korea; jchoe@kangwon.ac.kr; 3 Department of Medical Environmental Biology and Tropical Medicine, Kangwon National University School of Medicine, Chuncheon 24341, Republic of Korea; han.han@kangwon.ac.kr

**Keywords:** GABA aminotransferase, vigabatrin, epilepsy, GABAergic neurotransmission, anti-epileptic drugs, seizure control

## Abstract

Epilepsy affects approximately 50 million people worldwide, with nearly one-third of patients experiencing inadequate seizure control with conventional anti-epileptic drugs. The GABAergic system, responsible for inhibitory neurotransmission in the central nervous system, represents a critical target for seizure management. GABA aminotransferase (GABA-T), the enzyme responsible for GABA catabolism, has emerged as a particularly attractive therapeutic target. Inhibition of GABA-T increases synaptic GABA availability, enhancing inhibitory neurotransmission and raising the seizure threshold. Vigabatrin, an irreversible GABA-T inhibitor, has demonstrated remarkable efficacy in specific epilepsy syndromes, particularly infantile spasms and refractory partial seizures. However, its clinical utility is tempered by the risk of irreversible visual field defects, necessitating careful patient selection and monitoring. This review examines the molecular biology of GABA-T, the mechanisms of action of its inhibitors, clinical applications, safety considerations, and emerging developments in this therapeutic area. We discuss the structure–function relationships of GABA-T, the pharmacology of vigabatrin and experimental inhibitors, clinical efficacy across various epilepsy syndromes, adverse effect profiles, and future directions including novel inhibitors with improved safety profiles. Understanding the role of GABA-T in epilepsy pathophysiology and the therapeutic potential of its inhibitors provides insights into rational drug design and personalized treatment strategies for epilepsy management.

## 1. Introduction

Epilepsy is one of the most common serious neurological disorders, characterized by recurrent, unprovoked seizures resulting from abnormal, excessive, or synchronous neuronal activity in the brain [1,2,3,4]. According to the World Health Organization, epilepsy affects approximately 50 million individuals worldwide, imposing substantial burdens on patients, families, and healthcare systems [5,6]. Despite the availability of numerous anti-epileptic drugs (AEDs), approximately 30% of epilepsy patients continue to experience inadequate seizure control, defining the population with drug-resistant or refractory epilepsy [7,8].

The fundamental pathophysiology of epilepsy involves an imbalance between excitatory and inhibitory neurotransmission in the central nervous system (CNS) [9,10]. While glutamate serves as the primary excitatory neurotransmitter, γ-aminobutyric acid (GABA) functions as the principal inhibitory neurotransmitter in the mammalian brain. The GABAergic system plays a crucial role in modulating neuronal excitability, and its dysfunction has been implicated in various forms of epilepsy. Consequently, enhancing GABAergic inhibition represents a rational therapeutic strategy for seizure control [11,12,13].

Multiple approaches have been developed to enhance GABAergic neurotransmission, including direct GABA receptor agonists, modulators of GABA receptor function (such as benzodiazepines and barbiturates), inhibitors of GABA reuptake, and inhibitors of GABA metabolism. Among these strategies, the inhibition of GABA aminotransferase (GABA-T), the primary enzyme responsible for GABA catabolism, has proven particularly effective in specific clinical contexts [14,15,16].

GABA-T catalyzes the conversion of GABA to succinic semialdehyde, representing the rate-limiting step in GABA degradation [17,18]. By inhibiting this enzyme, GABA-T inhibitors increase GABA concentrations in the synaptic cleft and throughout the CNS, thereby enhancing inhibitory neurotransmission [19]. Vigabatrin (γ-vinyl-GABA), an irreversible GABA-T inhibitor, was developed in the 1970s and has since become an established treatment for specific epilepsy syndromes, particularly infantile spasms (West syndrome) and refractory complex partial seizures [20,21].

The clinical success of vigabatrin has validated GABA-T as a therapeutic target, yet its use has been limited by significant adverse effects, most notably irreversible bilateral concentric visual field defects [21,22]. This safety concern has motivated ongoing research into alternative GABA-T inhibitors with improved therapeutic indices, as well as refined patient selection criteria and monitoring strategies.

This comprehensive review examines the role of GABA-T in epilepsy pathophysiology and treatment. We begin by reviewing GABAergic neurotransmission and its relevance to epilepsy, then explore the molecular biology, structure, and function of GABA-T. Subsequently, we examine the pharmacology and clinical applications of GABA-T inhibitors, with particular emphasis on vigabatrin. We discuss the safety profile and monitoring requirements for these agents, genetic considerations affecting drug response, and emerging research directions. Finally, we consider the challenges and future prospects for GABA-T inhibition as an anti-epileptic strategy.

## 2. GABA and GABAergic Neurotransmission

### 2.1. The Central Role of GABA in Neuronal Inhibition

γ-Aminobutyric acid (GABA) is the predominant inhibitory neurotransmitter in the mammalian central nervous system, playing a fundamental role in regulating neuronal excitability and maintaining the balance between excitation and inhibition [23,24]. Discovered in 1950 by Eugene Roberts and Sam Frankel, GABA is present in high concentrations throughout the brain, with GABAergic neurons comprising approximately 20–30% of all cortical neurons [25,26]. The GABAergic system is essential for numerous physiological processes, including motor control, sensory perception, cognitive function, anxiety regulation, and sleep–wake cycles [27,28,29].

GABA mediates its inhibitory effects through two major classes of receptors: ionotropic GABA_A_ receptors and metabotropic GABA_B_ receptors [30]. GABA_A_ receptors are ligand-gated chloride channels that mediate fast inhibitory neurotransmission. Upon GABA binding, these receptors undergo conformational changes that open the chloride channel, allowing chloride ions to flow into the neuron [31,32]. This influx of negatively charged chloride ions hyperpolarizes the neuronal membrane, moving the membrane potential away from the threshold for action potential generation and thereby reducing neuronal excitability [33].

GABA_A_ receptors are heteromeric pentamers typically composed of two α subunits, two β subunits, and one γ subunit, though considerable subunit diversity exists [25,34]. This heterogeneity contributes to the pharmacological and functional diversity of GABAergic inhibition across brain regions. GABA_A_ receptors are also the primary targets for several important classes of psychoactive drugs, including benzodiazepines, barbiturates, neurosteroids, and certain anesthetics, which act as positive allosteric modulators to enhance GABAergic inhibition [25,35].

GABA_B_ receptors, in contrast, are G-protein-coupled receptors that mediate slower, prolonged inhibitory responses [36,37]. These receptors function as obligate heterodimers composed of GABA-B1 and GABA-B2 subunits [38,39,40]. Activation of GABA_B_ receptors leads to several downstream effects: activation of inwardly rectifying potassium channels (causing hyperpolarization), inhibition of voltage-gated calcium channels (reducing neurotransmitter release), and modulation of various intracellular signaling pathways [41,42]. GABA_B_ receptors are located both postsynaptically, where they mediate slow inhibitory postsynaptic potentials, and presynaptically, where they function as autoreceptors to regulate GABA release [30].

### 2.2. GABA Synthesis and Metabolism

**Figure 1:**
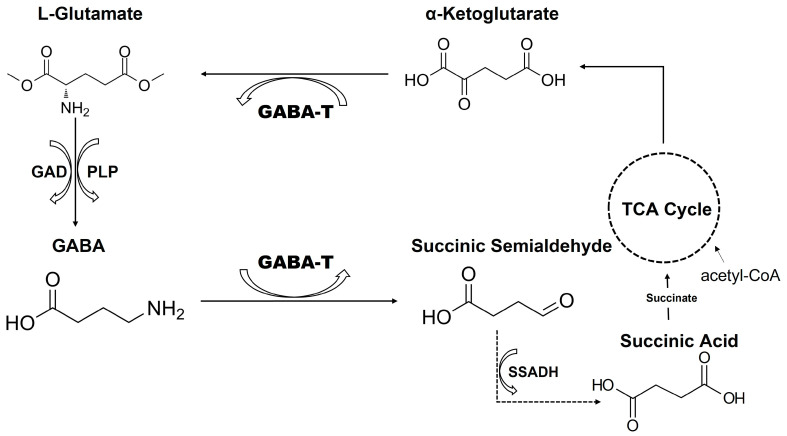
Schematic representation of the catalytic mechanism of GABA. Glutamic acid decarboxylase (GAD) converts L-glutamate to GABA using PLP (pyridoxal 5′-phosphate) as a cofactor. GABA is then metabolized by GABA aminotransferase (GABA-T) to succinic semialdehyde, which is subsequently oxidized by succinic semialdehyde dehydrogenase (SSADH) to succinic acid, linking GABA metabolism to the tricarboxylic acid (TCA) cycle.

GABA is synthesized from glutamate by the enzyme glutamic acid decarboxylase (GAD), which exists in two isoforms: GAD65 and GAD67. This reaction requires pyridoxal 5′-phosphate (vitamin B6) as a cofactor [43,44,45]. GAD65 is primarily localized to nerve terminals and is associated with vesicular GABA synthesis for neurotransmission, while GAD67 is distributed throughout the neuron and may contribute more to metabolic GABA pools [43,46,47,48]. The synthesis of GABA from glutamate represents a unique metabolic feature whereby the principal excitatory neurotransmitter is converted into the principal inhibitory neurotransmitter (Figure 1).

Following its release into the synaptic cleft and receptor binding, GABA is rapidly removed from the extracellular space by high-affinity GABA transporters (GATs). Four subtypes of GABA transporters have been identified in mammals: GAT-1, GAT-2, GAT-3, and the betaine/GABA transporter (BGT-1) [49,50]. GAT-1 is the predominant neuronal transporter and represents the primary target for GABA reuptake inhibitors such as tiagabine. These transporters are expressed on both neurons and astrocytes, enabling efficient clearance of synaptic GABA and termination of inhibitory signaling [51].

Once taken up into neurons or glia, GABA undergoes metabolic degradation primarily through the GABA shunt pathway, an alternative metabolic route that bypasses two steps of the tricarboxylic acid (TCA) cycle. The first and rate-limiting step of GABA catabolism is catalyzed by GABA aminotransferase (GABA-T) [52,53].

### 2.3. GABAergic Dysfunction in Epilepsy

The central role of γ-aminobutyric acid (GABA)-mediated inhibition in regulating neuronal excitability makes the GABAergic system a critical element in the pathogenesis of epilepsy [54]. Epileptic seizures arise when the balance between excitation and inhibition is disrupted, leading to excessive synchronous neuronal firing that overwhelms inhibitory control mechanisms [55]. Increasing evidence from clinical, genetic, and experimental studies has established that impairments in GABAergic signaling are a major contributor to seizure generation and epileptogenesis.

Clinical studies of epileptic brain tissue, particularly from patients with temporal lobe epilepsy (TLE), focal cortical dysplasia, and drug-resistant epilepsies, have identified multiple alterations in GABAergic markers [56,57,58,59]. These include reduced GABA concentrations, selective loss of GABAergic interneuron subtypes such as parvalbumin- and somatostatin-positive interneurons, and region-specific reductions in GABAergic synaptic density within the hippocampus, dentate gyrus, and temporal neocortex [58,60,61]. Changes in the expression and subunit composition of GABA_A_ and GABA_B_ receptors have also been documented, with several studies reporting downregulation of α1-containing GABA_A_ receptors and compensatory increases in α4-containing subunits, which exhibit altered pharmacological properties and reduced inhibitory strength. These receptor remodeling events may contribute to decreased seizure threshold and benzodiazepine resistance in chronic epilepsy [62,63].

Genetic evidence further supports a direct link between GABAergic dysfunction and seizure susceptibility. Mutations in genes encoding GABA_A_ receptor subunits, such as GABRA1, GABRB3, GABRG2, and GABRD, have been identified in patients with idiopathic generalized epilepsies, Dravet syndrome, childhood absence epilepsy, and febrile seizures [64,65]. Many of these mutations result in reduced receptor trafficking to the membrane, impaired channel gating, or dominant-negative effects that substantially diminish inhibitory tone. Additionally, variants in genes encoding GABA transporters (e.g., SLC6A1) and enzymes involved in GABA metabolism have broadened recognition of genetic GABAergic defects in epileptogenesis.

Experimental models have provided further mechanistic insights. Pharmacological blockade of GABA_A_ receptors using bicuculline, picrotoxin, or PTZ reliably induces seizures across species, confirming the necessity of intact GABAergic inhibition for maintaining normal neuronal activity [66,67]. Conversely, many clinically effective antiseizure medications, including benzodiazepines, barbiturates, vigabatrin, and tiagabine, act by enhancing GABAergic signaling, underscoring the therapeutic relevance of this pathway [68]. Genetic mouse models with targeted disruption of GABA_A_ receptor subunits or interneuron development pathways (e.g., Arx, Dlx, or Gabrb3 knockout lines) frequently exhibit spontaneous seizures and behavioral phenotypes analogous to human epileptic syndromes, reinforcing the causal role of impaired inhibition [69,70].

The temporal dynamics of GABAergic changes in epilepsy are complex. Acute seizures may transiently enhance GABA release or upregulate certain GABA receptor subunits as an adaptive response, whereas chronic epilepsy is commonly associated with interneuron loss, impaired GABA synthesis, deficits in chloride homeostasis (e.g., NKCC1/KCC2 imbalance), and synaptic reorganization that collectively reduce inhibitory efficacy. Such long-term alterations contribute to hyperexcitable circuits capable of generating spontaneous recurrent seizures [54].

Importantly, different forms of epilepsy may involve distinct mechanisms of GABAergic dysfunction. For example, Temporal lobe epilepsy (TLE): marked loss of hippocampal interneurons, mossy fiber sprouting, and altered δ-subunit-containing GABA_A_ receptors; Absence epilepsy: disturbances in thalamocortical oscillations involving enhanced GABA_B_ receptor-mediated inhibition and altered tonic GABA currents in thalamic relay neurons; Focal cortical dysplasia: disrupted interneuron migration and abnormal lamination result in spatially heterogeneous inhibitory deficits; and Neonatal seizures: immature chloride transporter expression renders GABA depolarizing rather than inhibitory, contributing to seizure susceptibility [71]. Understanding these distinct patterns of GABAergic impairment is crucial for the rational design of targeted therapies. Emerging research aims to develop strategies that restore inhibitory balance, including interneuron transplantation, modulation of chloride transport, and selective targeting of GABA metabolic enzymes such as GABA-T.

### 2.4. Therapeutic Implications

The central role of GABA in controlling neuronal excitability has made the GABAergic system a major target for anti-epileptic drug development. Multiple mechanisms for enhancing GABAergic inhibition have been successfully exploited therapeutically. These include positive allosteric modulation of GABA_A_ receptors (benzodiazepines, barbiturates), inhibition of GABA reuptake (tiagabine), and inhibition of GABA catabolism (vigabatrin) [72,73,74,75].

Each approach has distinct advantages and limitations. Positive allosteric modulators of GABA_A_ receptors are highly effective for acute seizure control but are associated with tolerance, dependence, and sedation [68,76]. GABA reuptake inhibitors increase synaptic GABA availability but may have limited efficacy due to compensatory mechanisms. GABA-T inhibitors offer the advantage of increasing GABA levels throughout all compartments of the CNS, potentially providing broader therapeutic effects [77].

The success of GABAergic AEDs in clinical practice validates this therapeutic strategy while highlighting the importance of developing agents with improved selectivity, efficacy, and safety profiles. Understanding the detailed molecular mechanisms of GABAergic neurotransmission and its metabolism continues to inform drug discovery efforts aimed at optimizing seizure control while minimizing adverse effects.

## 3. GABA Aminotransferase: Structure, Function, and Mechanism

### 3.1. Molecular Structure and Organization

GABA aminotransferase (GABA-T; EC 2.6.1.19), also known as γ-aminobutyrate:α-ketoglutarate aminotransferase, is a pyridoxal 5′-phosphate (PLP)-dependent enzyme that catalyzes the initial step in GABA catabolism (Table 1). The enzyme is encoded by the ABAT gene in humans, located on chromosome 16p13.2. The gene consists of 16 exons and spans approximately 100 kilobases of genomic DNA [52,78,79].

GABA-T is a homodimeric protein with a molecular mass of approximately 109 kDa (54.5 kDa per monomer). Crystal structures of GABA-T from various species, including pig, mouse, and bacterial sources, have been solved, providing detailed insights into its three-dimensional architecture. Each monomer consists of approximately 500 amino acids and adopts a characteristic fold found in PLP-dependent aminotransferases [80].

The structure of GABA-T can be divided into two distinct domains: a large domain containing the active site and a small domain involved in dimerization and substrate binding. The large domain displays a typical α/β architecture with a central β-sheet surrounded by α-helices. The active site is located at the interface between the two domains and is formed by residues from both monomers of the homodimer, explaining the functional importance of the dimeric quaternary structure [25,52].

**Table 1:** Structural and Functional Characteristics of GABA-T.

Feature	Description	References
Enzyme name	GABA aminotransferase (GABA-T); EC 2.6.1.19	[80,81]
Gene	ABAT (chromosome 16p13.2, 16 exons)	
Molecular mass	~109 kDa (homodimer; ~54.5 kDa per monomer)	
Amino acid residues	~500 amino acids per monomer	
Domain structure	Large catalytic domain (residues 48–374, active site Lys329); small domain (residues 1–47 and 375–500) involved in dimerization and substrate binding	
Cofactor	Pyridoxal 5′-phosphate (PLP) covalently bound to Lys329 via Schiff base	
Substrate specificity	Prefers GABA and α-ketoglutarate; catalyzes formation of succinic semialdehyde and glutamate	
Subcellular localization	Mitochondrial matrix (post-cleavage of targeting sequence)	
Physiological function	Catalyzes rate-limiting step in GABA degradation; links neurotransmitter metabolism to energy production	

**Figure 2:**
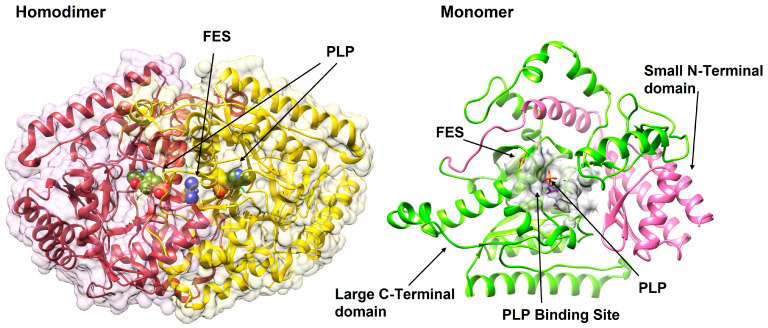
The 3D structural overview of GABA-T (PDB ID: 1OHW). The enzyme exists as a functional homodimer (**left**), with each monomer (**right**) comprising a large C-terminal catalytic domain (green) and a small N-terminal domain involved in dimerization and substrate binding (pink). The PLP cofactor and FES (2Fe-2S; iron-sulfur cluster), an intrinsic cofactor, are shown at the active site within the PLP-binding pocket (grey).

The PLP cofactor is covalently bound to a conserved lysine residue (Lys329 in human GABA-T) through a Schiff base linkage, forming an internal aldimine. The PLP-binding site is highly conserved among aminotransferases and includes residues that properly orient and stabilize the cofactor [82,83,84]. The pyridine ring of PLP is held in position through interactions with aromatic residues and hydrogen bonding networks, ensuring optimal geometry for catalysis (Figure 2).

A distinctive feature of GABA-T is its relatively narrow substrate-binding pocket, which accommodates the short, four-carbon structure of GABA while excluding larger amino acids [85]. Key residues lining the substrate-binding site include Glu270, Arg192, and Phe189, which interact with the amino and carboxyl groups of GABA. This specificity is crucial for the enzyme’s physiological function and has important implications for inhibitor design.

### 3.2. Catalytic Mechanism

GABA-T catalyzes the reversible transamination of GABA and α-ketoglutarate to produce succinic semialdehyde and glutamate. The reaction proceeds through the classic ping-pong bi-bi mechanism characteristic of PLP-dependent aminotransferases, involving two half-reactions [86,87,88].

In the first half-reaction, GABA binds to the active site and forms an external aldimine with the PLP cofactor, displacing the lysine residue. A catalytic base (typically Lys329) abstracts the α-proton from GABA, and the resulting carbanion intermediate is stabilized by the electron-withdrawing PLP system. The Schiff base then undergoes transamination, releasing succinic semialdehyde and leaving the PLP in its pyridoxamine 5′-phosphate (PMP) form, with the amino group from GABA now attached to the cofactor. In the second half-reaction, α-ketoglutarate binds to the active site and reacts with PMP to form a Schiff base. Through the reverse sequence of steps, the amino group is transferred from PMP to α-ketoglutarate, producing glutamate and regenerating the PLP-enzyme complex. The enzyme is now ready for another catalytic cycle [80].

The catalytic efficiency of GABA-T is impressive, with kcat values ranging from 10 to 50 s^−1^ and Km values for GABA typically between 1 and 7 mM, depending on the species and experimental conditions. The enzyme shows high specificity for GABA as the amino donor, though it can also accept β-alanine at much lower efficiency. For the amino acceptor, α-ketoglutarate is strongly preferred, although other α-keto acids can serve as substrates at reduced rates [80,89].

### 3.3. Cellular Localization and Physiological Function

A critical aspect of GABA-T biology is its subcellular localization. The enzyme is localized to the mitochondrial matrix, where it associates with the outer surface of the inner mitochondrial membrane. This localization is directed by an N-terminal mitochondrial targeting sequence that is cleaved upon import into mitochondria. The mature, active form of GABA-T lacks this targeting peptide [90,91].

The mitochondrial localization of GABA-T has several important implications. First, it couples GABA metabolism directly to cellular energy production, as the product succinic semialdehyde is further metabolized to succinate, which enters the tricarboxylic acid cycle. This integration of neurotransmitter metabolism with energy metabolism may have important regulatory functions [91].

Second, the compartmentalization of GABA-T means that GABA must be transported into mitochondria before degradation can occur. This transport process represents an additional regulatory point in GABA homeostasis. The mitochondrial GABA transporter has not been fully characterized, but its activity may influence the rate of GABA catabolism [91,92,93].

Third, the mitochondrial location affects the pharmacokinetics and pharmacodynamics of GABA-T inhibitors. Inhibitors must cross not only the plasma membrane but also the mitochondrial membranes to reach their target enzyme. This requirement has influenced inhibitor design strategies [94].

GABA-T is expressed throughout the brain, with particularly high levels in regions rich in GABAergic neurons, including the cerebral cortex, hippocampus, cerebellum, basal ganglia, and hypothalamus. The enzyme is present in both neurons and astrocytes, reflecting the distributed nature of GABA metabolism. However, the relative contributions of neuronal versus glial GABA-T to overall GABA catabolism remain incompletely understood [95].

### 3.4. GABA-T in the GABA Shunt Pathway

**Figure 3:**
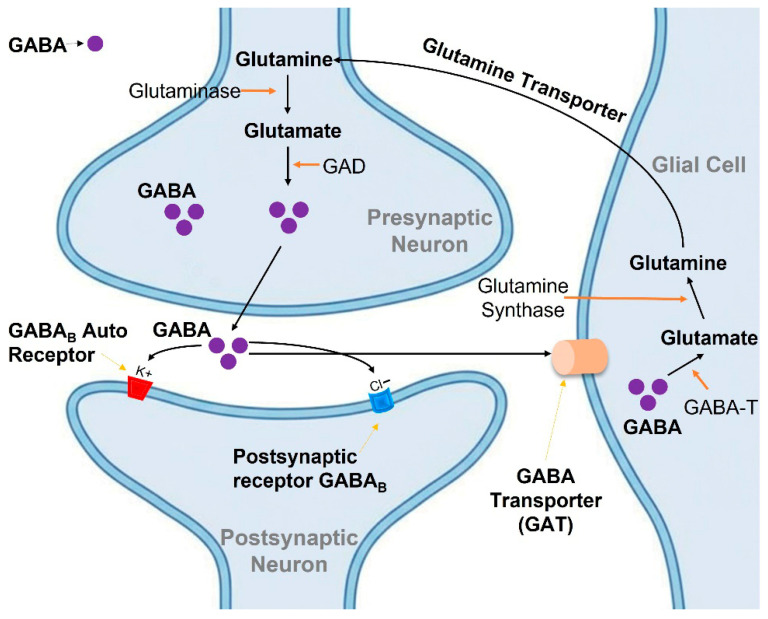
Schematic illustration of GABAergic neurotransmission and metabolism in the GABA shunt pathway. GABA is synthesized from glutamate by glutamic acid decarboxylase (GAD) in the presynaptic neuron and released into the synaptic cleft to activate GABA_B_ receptors. GABA is then taken up by glial cells and neurons via GABA transporters (GAT) and metabolized by GABA-T, with the resulting glutamate converted to glutamine and recycled back to neurons.

The GABA shunt represents an alternative route for metabolizing α-ketoglutarate to succinate, bypassing two steps of the citric acid cycle. This pathway consists of three enzymes: glutamate decarboxylase (GAD), GABA-T, and succinic semialdehyde dehydrogenase (SSADH). The net reaction of the shunt converts α-ketoglutarate and glutamate to succinate and GABA, with no net production of ATP or reducing equivalents in the bypass portion (Figure 3) [86,88,91,96].

The physiological significance of the GABA shunt has been debated. While initially considered primarily a neurotransmitter metabolism pathway, evidence suggests the shunt may play important roles in cellular energy metabolism, particularly under conditions of metabolic stress. The shunt can function as an anaplerotic pathway, replenishing TCA cycle intermediates, and may help maintain the NADH/NAD^+^ ratio under certain conditions [97,98].

Importantly, the GABA shunt provides a metabolic link between GABAergic neurotransmission and cellular bioenergetics. Alterations in either neurotransmitter function or energy metabolism can therefore affect the other. This coupling may be particularly relevant in epilepsy, where both excessive neuronal activity and metabolic dysfunction can contribute to pathophysiology [93,94,99].

### 3.5. GABA-T Regulation, Expression, and Enzymatic Activity

The expression of GABA-T is regulated at multiple levels, including transcriptional control, post-transcriptional modifications, and post-translational regulation. The ABAT gene promoter contains binding sites for several transcription factors, allowing for tissue-specific and developmentally regulated expression [100,101]. GABA-T expression shows developmental changes, generally increasing during postnatal brain maturation. This developmental pattern parallels the maturation of GABAergic systems and may contribute to age-dependent differences in seizure susceptibility and response to GABAergic drugs [102,103,104].

Several studies have examined factors that regulate GABA-T expression. Neuronal activity, hormones, and metabolic status can all influence GABA-T levels. For example, chronic seizure activity has been reported to alter GABA-T expression in some brain regions, potentially representing a compensatory response to altered GABA metabolism [105].

Post-translational modifications of GABA-T, including phosphorylation, may modulate enzyme activity, though these regulatory mechanisms are not fully characterized. The stability of the GABA-T protein and its turnover rate in cells are areas requiring further investigation.

### 3.6. Pathological Alterations in GABA-T Function

While increased GABA-T activity has been hypothesized to contribute to epilepsy by accelerating GABA catabolism, direct evidence for this mechanism in human epilepsy is limited. More commonly, alterations in GABA-T expression or activity in epilepsy appear to be secondary consequences of seizure activity rather than primary causative factors.

Conversely, loss-of-function mutations in the ABAT gene cause GABA-T deficiency, a rare autosomal recessive neurometabolic disorder. This condition is characterized by psychomotor retardation, hypotonia, hyperreflexia, seizures, and accelerated linear growth [106,107]. The paradoxical occurrence of seizures in patients with GABA-T deficiency, despite presumed elevation of GABA levels, remains incompletely explained but may relate to altered neuronal development, receptor downregulation, or other compensatory mechanisms.

The study of GABA-T deficiency has provided insights into the physiological roles of this enzyme beyond simple GABA catabolism [86,108]. The diverse clinical manifestations of GABA-T deficiency suggest that the enzyme plays important roles in brain development, metabolic regulation, and perhaps other cellular processes.

## 4. GABA-T as a Therapeutic Target in Epilepsy

### 4.1. Rationale for GABA-T Inhibition

The concept of inhibiting GABA-T to enhance GABAergic neurotransmission and thereby suppress seizures is based on solid theoretical foundations. By blocking the primary catabolic pathway for GABA, GABA-T inhibitors increase GABA concentrations throughout the brain [93], including in the synaptic cleft, neuronal cytoplasm, and glial cells. This broad elevation of GABA levels enhances inhibitory tone across neural networks, counteracting the excessive excitability characteristic of epilepsy.

Several features make GABA-T an attractive target for anti-epileptic therapy. First, GABA-T inhibition affects GABA metabolism systemically throughout the CNS rather than modulating individual synapses, potentially providing more comprehensive seizure control [77]. Second, unlike direct receptor agonists, GABA-T inhibitors work by preserving endogenously released GABA, maintaining the spatial and temporal specificity of physiological GABAergic signaling while amplifying its magnitude [17]. Third, GABA-T inhibition may be less likely to cause receptor desensitization or tolerance compared to direct receptor activation [109], though clinical experience suggests some tolerance can still develop.

The elevation of GABA levels following GABA-T inhibition has been convincingly demonstrated through multiple experimental approaches. Magnetic resonance spectroscopy (MRS) studies in humans have shown substantial increases in brain GABA concentrations following vigabatrin treatment, with increases of 100–200% commonly observed [110,111]. These elevations correlate with the degree of GABA-T inhibition and persist for extended periods due to the irreversible nature of vigabatrin’s mechanism.

Animal studies have similarly demonstrated elevated GABA levels in brain tissue, cerebrospinal fluid, and extracellular fluid following GABA-T inhibition [112,113]. Microdialysis studies have shown increased basal GABA levels and enhanced GABA release following neuronal stimulation in vigabatrin-treated animals [114]. These findings confirm that GABA-T inhibition effectively increases GABA availability for neurotransmission.

### 4.2. Effects on Seizure Threshold and Epileptic Activity

Extensive preclinical studies in animal models have validated the anti-epileptic efficacy of GABA-T inhibition [115,116]. Vigabatrin and other GABA-T inhibitors demonstrate broad-spectrum activity in acute chemical seizure models induced by pentylenetetrazol, picrotoxin, and bicuculline, where elevated extracellular GABA effectively counteracts the reduced inhibition produced by GABA_A_ receptor antagonists, resulting in increased seizure threshold and attenuation of seizure severity [117,118]. Similar effects are observed in electrical seizure models, including maximal electroshock and kindling paradigms, where GABA-T inhibitors reliably raise seizure threshold, reduce after discharge duration, and suppress seizure generalization [68,116,119]. In kindling models that recapitulate progressive aspects of epileptogenesis, vigabatrin has been shown to slow kindling acquisition and delay secondary generalization, indicating potential disease-modifying effects rather than merely symptomatic seizure suppression [120].

Genetic models of epilepsy provide important insights into syndrome-specific responses to GABA-T inhibition. In mice carrying mutations in GABRA1 or GABRG2, which model generalized epilepsies associated with impaired GABA_A_ receptor function, vigabatrin markedly reduces seizure frequency and improves survival, likely by compensating for reduced synaptic inhibition through sustained increases in extracellular GABA [121,122]. In the absence of epilepsy models such as Stargazer and Tottering mice, vigabatrin decreases spike-and-wave discharges, though responsiveness is strain-dependent and influenced by thalamocortical GABAergic mechanisms [123]. Conversely, models such as SCN1A mutant mice (Dravet syndrome) demonstrate limited or variable responses to GABA-T inhibition because their underlying pathology reflects sodium channel dysfunction rather than primary deficits in GABAergic inhibition [119]. These findings underscore that GABA-T inhibitors are most effective in epilepsy types where impaired GABAergic signaling is a central pathogenic mechanism.

Mechanistic studies using hippocampal slices and neuronal cultures show that GABA-T inhibition enhances both phasic and tonic GABAergic inhibition. Elevation of tonic inhibitory currents, mediated by high-affinity extrasynaptic GABA_A_ receptors containing δ subunits, appears especially important for reducing network excitability because tonic inhibition provides a persistent, non-desensitizing inhibitory influence on neuronal firing [77,112,124,125]. Electrophysiological recordings further demonstrate that GABA-T inhibition prolongs inhibitory postsynaptic currents, elevates ambient GABA levels, decreases population spike amplitudes, and suppresses epileptiform burst activity in hyperexcitable hippocampal networks [14,126,127,128]. These cellular and network-level effects collectively support the conclusion that GABA-T inhibitors help restore a more physiological balance between excitation and inhibition. Rather than indiscriminately suppressing all neural activity, GABA-T inhibition selectively enhances inhibitory tone in overactive circuits, thereby normalizing firing patterns and reducing pathological synchronization.

### 4.3. Advantages over Alternative GABAergic Strategies

Compared to other approaches for enhancing GABAergic function, GABA-T inhibition offers several potential advantages. Unlike benzodiazepines and barbiturates, which directly enhance GABA_A_ receptor function, GABA-T inhibitors work by increasing the concentration of endogenous GABA [129,130]. This mechanism preserves the normal spatial and temporal patterns of GABAergic signaling, potentially reducing side effects related to excessive, non-physiological receptor activation.

GABA-T inhibitors differ from GABA reuptake inhibitors in their site of action. While reuptake inhibitors like tiagabine primarily increase GABA concentrations in the synaptic cleft, GABA-T inhibitors elevate GABA throughout all cellular compartments [93,131]. This broader distribution may provide advantages in some clinical contexts, though it may also contribute to adverse effects.

The irreversible mechanism of vigabatrin produces long-lasting GABA elevation even after the drug is cleared from the body. This extended duration of action means that once-daily or even less frequent dosing may be sufficient for seizure control [132,133,134]. However, this irreversibility also means that adverse effects may persist long after drug discontinuation. GABA-T inhibitors may have effects beyond the simple elevation of GABA levels. Some evidence suggests that chronic GABA-T inhibition may induce compensatory changes in GABAergic systems, including alterations in GABA receptor expression or function [134]. These adaptive responses may contribute to both therapeutic effects and the development of tolerance or adverse effects.

### 4.4. Limitations and Considerations

Despite the strong rationale for GABA-T inhibition, several limitations must be acknowledged. First, the paradox of GABA-T deficiency, in which genetic absence of GABA-T leads to seizures rather than seizure protection [60,68], suggests that the relationship between GABA levels and seizure control is complex. Excessive GABA elevation during critical developmental periods may disrupt normal brain maturation, and chronic supraphysiological GABA levels may trigger compensatory downregulation of inhibitory function [135,136]. Second, GABA-T inhibition affects GABA metabolism throughout the body, not just in epileptic foci. This systemic effect may contribute to adverse effects unrelated to seizure control. The lack of regional or circuit-specific selectivity limits the therapeutic index of current GABA-T inhibitors. Third, individual variability in response to GABA-T inhibitors is substantial, with some patients showing excellent seizure control while others derive minimal benefit [137]. The molecular basis for this variability is incompletely understood but likely involves differences in epilepsy etiology, GABAergic system function, and pharmacogenomic factors. Fourth, the development of tolerance to GABA-T inhibitors has been observed in some patients, potentially limiting long-term efficacy [138,139]. The mechanisms underlying tolerance development, whether pharmacokinetic adaptation, pharmacodynamic compensation, or disease progression, require further elucidation.

Despite these limitations, the clinical success of vigabatrin in specific epilepsy syndromes has validated GABA-T as a therapeutic target. Ongoing research aims to develop next-generation GABA-T inhibitors that retain efficacy while overcoming the limitations of current agents, particularly the risk of visual field defects.

## 5. Molecular and Genetic Considerations

### 5.1. GABA-T Gene Mutations and Deficiency Phenotypes

Loss-of-function mutations in the ABAT gene, which encodes GABA transaminase (GABA-T), cause an autosomal recessive disorder known as GABA-T deficiency. Fewer than a hundred cases have been reported, typically presenting in infancy with severe psychomotor retardation, hypotonia, seizures, movement disorders, and variable hepatic dysfunction.

Distinctive systemic findings include accelerated linear growth, advanced bone age, and elevated growth hormone and IGF-1 levels. Neuroimaging often reveals cerebral atrophy, delayed myelination, and hyperintense T2 signals in the globus pallidus and thalamus. Biochemically, cerebrospinal fluid (CSF) GABA concentrations are markedly elevated, up to twentyfold higher than normal, accompanied by increases in β-alanine and homocarnosine [140,141].

### 5.2. Genetic Spectrum and Mutation Types

Pathogenic ABAT variants include nonsense, missense, frameshift, and splice-site mutations, with most patients being compound heterozygotes. These mutations disrupt enzyme function, but genotype–phenotype correlations remain unclear due to the rarity and heterogeneity of reported cases [142].

### 5.3. The GABA-T Deficiency Paradox

Despite profound elevations in GABA levels, many GABA-T-deficient patients experience seizures, a paradox that challenges the assumption that increased GABA is uniformly protective. Proposed mechanisms include developmental interference of excess GABA on neuronal maturation and circuit formation, compensatory downregulation of GABA receptors, mitochondrial dysfunction due to disruption of the GABA shunt, and altered chloride homeostasis leading to excitatory GABAergic signaling during early brain development. Structural brain abnormalities seen in these patients may also contribute to epileptogenesis. This paradox underscores the context-dependent nature of GABAergic neurotransmission [143,144].

### 5.4. Pharmacogenomics of GABA-T Inhibitor Response

Although routine pharmacogenomic testing is not yet integrated into clinical use of vigabatrin, genetic factors may influence both efficacy and toxicity. Variations within ABAT could modify baseline enzyme activity, vigabatrin binding affinity, or enzyme resynthesis rate following inactivation [78]. Additionally, polymorphisms in other GABAergic system genes, including GABA_A_ receptor subunits (e.g., *GABRA1*, *GABRB3*, *GABRG2*), glutamic acid decarboxylase (*GAD1*, *GAD2*), and GABA transporters (*SLC6A1*), may modulate the drug’s pharmacodynamic effects [145,146].

### 5.5. Genetic Factors and Retinal Toxicity

Understanding genetic susceptibility to vigabatrin-induced visual field defects remains a key unmet need. Candidate genes under investigation include those involved in retinal oxidative stress defense, taurine metabolism, GABA receptor signaling, and mitochondrial bioenergetics [147]. However, no validated genetic markers predicting retinal toxicity have yet been identified.

### 5.6. Epilepsy-Associated Gene Interactions

The genetic background of the underlying epilepsy may also influence vigabatrin response. Its exceptional efficacy in tuberous sclerosis complex (TSC), related infantile spasms suggests that TSC1/TSC2 pathway dysregulation may enhance responsiveness to GABAergic modulation [148]. Conversely, in SCN1A-related epilepsies such as Dravet syndrome, vigabatrin’s effects are variable. Mutations in GABA_A_ receptor genes may similarly alter drug response [149], though supporting data remain limited.

## 6. GABA-T Inhibitors: Development and Mechanisms

### 6.1. Vigabatrin (γ-vinyl-GABA)

#### 6.1.1. Discovery and Development History

Vigabatrin, chemically known as γ-vinyl-GABA or 4-amino-5-hexenoic acid, represents one of the most successful examples of rational drug design based on enzyme mechanism [150,151]. The compound was first synthesized in the 1970s by researchers at Merrell Dow Pharmaceuticals as part of a program aimed at developing irreversible inhibitors of GABA-T [152,153]. The design strategy was based on the concept of mechanism-based enzyme inactivation, also known as “suicide inhibition” [154].

The structural design of vigabatrin was inspired by the observation that vinyl-substituted amino acids could serve as mechanism-based inhibitors of PLP-dependent enzymes [155]. The molecule consists of GABA with a vinyl group attached to the γ-carbon, creating a structural analog that undergoes the initial steps of normal catalysis but then irreversibly inactivates the enzyme. Initial preclinical studies in the late 1970s demonstrated potent GABA-T inhibition, substantial increases in brain GABA levels, and broad-spectrum anti-seizure activity in animal models [156,157,158,159].

Clinical development of vigabatrin began in the early 1980s [160]. The drug was first approved in the United Kingdom in 1989 as adjunctive therapy for refractory epilepsy [161,162,163]. Subsequently, vigabatrin received regulatory approval in numerous countries throughout Europe, South America, Asia, and other regions. However, approval in the United States was delayed by safety concerns, particularly regarding retinal toxicity observed in preclinical studies and visual field defects reported in clinical trials [161].

The recognition of vigabatrin’s particular efficacy in infantile spasms (West syndrome) emerged from clinical observations in the 1990s. This indication became especially important because infantile spasms are notoriously difficult to treat, and vigabatrin demonstrated response rates superior to many alternative therapies. Based on this evidence, the US Food and Drug Administration (FDA) ultimately approved vigabatrin in 2009 for two specific indications: infantile spasms and refractory complex partial seizures in adults, with a Risk Evaluation and Mitigation Strategy (REMS) program due to the risk of permanent vision loss [164,165].

#### 6.1.2. Mechanism of Irreversible Inhibition

Vigabatrin functions as a mechanism-based irreversible inhibitor, or “suicide substrate,” of GABA-T. This type of inhibition represents an elegant pharmacological strategy in which the inhibitor molecule is a substrate analog that undergoes the initial steps of normal enzyme catalysis. However, during the catalytic process, a highly reactive intermediate is generated that forms a covalent bond with the enzyme, resulting in permanent inactivation [166,167].

**Figure 4:**
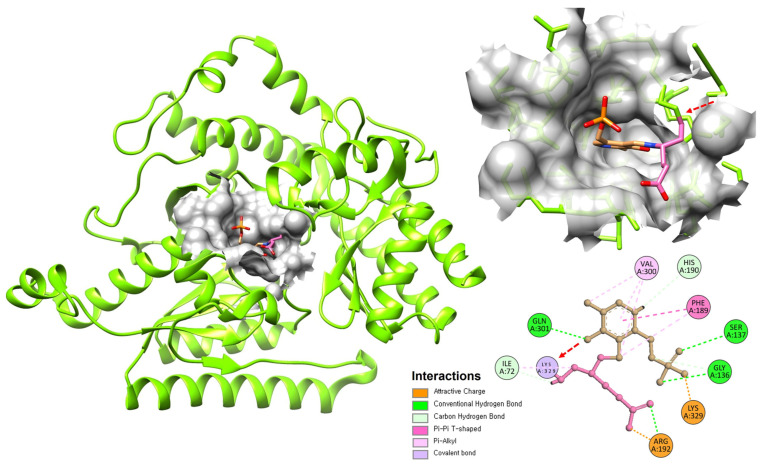
Binding interaction of GABA-T monomer with PLP and Tiagabine. The structure shows PLP (Pink) and Tiagabine (Brown) bound at the active site of GABA-T. A covalent bond between PLP and Lys329 is highlighted by the red arrow, with the 2D interaction map showing additional hydrogen bonding and hydrophobic interactions stabilizing the complex.

The mechanism of vigabatrin’s action begins with the drug entering the GABA-T active site and binding to the PLP cofactor, forming an external aldimine similar to what occurs with the natural substrate GABA. The catalytic base then abstracts the α-proton from vigabatrin, forming a carbanion intermediate stabilized by the PLP system. At this point, the mechanism diverges from normal GABA metabolism. The vinyl group on vigabatrin is positioned such that it can participate in Michael addition chemistry. Following formation of the carbanion, the activated intermediate undergoes an internal rearrangement in which the double bond of the vinyl group becomes conjugated with the PLP system. This creates an α,β-unsaturated imine, a highly electrophilic species capable of reacting with nucleophilic residues in the active site. A nucleophilic amino acid residue in the active site (likely a lysine or cysteine) then attacks the activated intermediate, forming a covalent adduct. This covalent modification irreversibly inactivates the enzyme (Figure 4). Crystallographic studies have confirmed the formation of stable covalent complexes between vigabatrin-derived intermediates and GABA-T. The specific residues modified and the exact structure of the covalent adduct have been characterized through X-ray crystallography and mass spectrometry studies [20,168,169].

The irreversibility of vigabatrin’s inhibition has profound pharmacological implications. Once GABA-T is inactivated, the enzyme cannot be reactivated. Recovery of GABA-T activity depends entirely on the synthesis of new enzyme protein, a process that takes several days to weeks. This means that the duration of vigabatrin’s pharmacodynamic effect far exceeds its pharmacokinetic half-life in plasma. Even after vigabatrin is eliminated from the body (typically within 24–48 h), GABA levels remain elevated and seizure control persists [80,170].

Vigabatrin is administered as a racemic mixture of S(+) and R(−) enantiomers. The S(+) enantiomer is the active form and is approximately 200-fold more potent as a GABA-T inhibitor than the R(−) enantiomer. However, both enantiomers are absorbed and distributed similarly, and the inactive R(−) enantiomer may contribute to some of the drug’s adverse effects [171,172,173]. The development of enantiopure formulations was explored but ultimately not pursued commercially.

#### 6.1.3. Pharmacokinetics and Pharmacodynamics

Vigabatrin exhibits favorable pharmacokinetic properties that contribute to its clinical utility. The drug is administered orally and is rapidly and almost completely absorbed from the gastrointestinal tract, with peak plasma concentrations typically achieved within 1–2 h after dosing. Bioavailability is approximately 60–80% and is not significantly affected by food, though meals may slightly delay absorption [174,175].

Vigabatrin displays linear pharmacokinetics across the therapeutic dose range, with dose-proportional increases in plasma concentrations. The volume of distribution is approximately 0.8 L/kg, suggesting distribution throughout total body water with minimal tissue binding. Importantly, vigabatrin readily crosses the blood–brain barrier, achieving brain concentrations comparable to plasma levels. This efficient CNS penetration is essential for the drug’s therapeutic action [168].

One of the most distinctive pharmacokinetic features of vigabatrin is its lack of significant protein binding. Approximately 95–99% of vigabatrin in plasma is in the free, unbound form. This characteristic minimizes the potential for protein-binding displacement interactions with other drugs and ensures predictable free drug concentrations [176].

Vigabatrin undergoes minimal metabolism, with approximately 95% of an administered dose excreted unchanged in urine. The small amount of metabolism that occurs produces inactive products and does not involve cytochrome P450 enzymes. This lack of hepatic metabolism has important clinical implications: vigabatrin does not induce or inhibit drug-metabolizing enzymes, resulting in minimal pharmacokinetic interactions with other medications. This is a significant advantage in epilepsy treatment, where polypharmacy is common [177]. The elimination half-life of vigabatrin from plasma is approximately 5–8 h in adults, with renal clearance being the primary route of elimination. In patients with impaired renal function, vigabatrin clearance is reduced proportionally to the decrease in creatinine clearance, necessitating dose adjustments. Age also affects pharmacokinetics: elimination is slower in elderly patients (half-life 12–13 h) and faster in children (half-life 4–6 h), requiring age-appropriate dosing strategies [178]. Despite the relatively short plasma half-life, the pharmacodynamic effects of vigabatrin are prolonged due to irreversible enzyme inhibition. Studies using PET imaging and biochemical markers have shown that GABA-T activity remains suppressed for weeks after vigabatrin discontinuation. Brain GABA levels, measured by magnetic resonance spectroscopy, remain elevated for 4–8 weeks after stopping treatment, gradually returning to baseline as new GABA-T enzyme is synthesized [179].

This dissociation between pharmacokinetics and pharmacodynamics has clinical implications for dosing strategies. Vigabatrin is typically administered once or twice daily, and steady-state GABA elevation is achieved within several days. When discontinuing vigabatrin, gradual tapering is recommended not because of withdrawal effects related to drug elimination, but rather to minimize the risk of seizure exacerbation as GABA levels gradually decline.

### 6.2. Development of Second-Generation Inhibitors

The proven efficacy of vigabatrin, coupled with its risk of visual toxicity, has prompted extensive research into safer and more selective GABA-T inhibitors. Current strategies focus on optimizing pharmacodynamic and pharmacokinetic properties to maintain efficacy while reducing adverse effects. Efforts include developing reversible inhibitors, prodrug formulations, and modified GABA analogs that enhance central nervous system (CNS) selectivity and minimize peripheral exposure.

Unlike vigabatrin’s irreversible mechanism, reversible inhibitors interact non-covalently with GABA-T, allowing temporary enzyme inhibition (Table 2). New strategies involve developing reversible GABA-T inhibitors, such as cyclic GABA analogs and ethanolamine O-sulfate, which interact non-covalently with the enzyme [180,181,182,183]. These compounds offer potential benefits such as dose flexibility, predictable pharmacokinetics, and faster recovery after treatment cessation. Although they have shown modest activity in preclinical models, achieving sufficient potency and brain penetration remains challenging.

**Figure 5:**
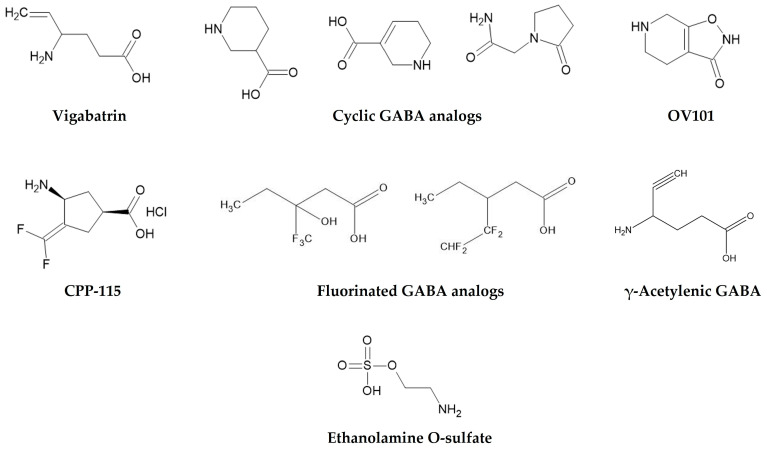
Chemical structures of vigabatrin and representative GABA-T inhibitors, including cyclic, fluorinated, acetylenic GABA analogs and ethanolamine O-sulfate.

CPP-115, a rationally designed analog of vigabatrin (Figure 5), represents a significant advancement in this direction. With enhanced lipophilicity and improved CNS selectivity, CPP-115 achieves greater central GABA-T inhibition at lower systemic concentrations, theoretically reducing retinal exposure. Preclinical results are promising, but clinical development remains in its early stages, and further evaluation is needed to confirm its safety and efficacy [158,184,185,186]. Similarly, γ-acetylenic GABA, another mechanism-based inactivator, has shown high in vitro potency, though its safety has not yet been established.

Beyond direct enzyme inhibition, alternative pharmacological strategies have been explored. OV101 (Gaboxadol), a selective agonist at extrasynaptic GABA_A_ receptors containing δ-subunits, enhances tonic inhibition independently of GABA-T. Initially developed for insomnia, Gaboxadol was later evaluated for epilepsy, Angelman syndrome, and Fragile X syndrome, but clinical results were mixed, and development for epilepsy was discontinued [187,188]. Although its mechanism is distinct from GABA-T inhibition, OV101 illustrates how enhancing tonic GABAergic inhibition can be achieved at the receptor level, providing a useful comparator for GABA-T-targeting approaches.

Additionally, fluorinated GABA analogs have been designed to modulate lipophilicity, enzyme affinity, and metabolic stability, with some analogs showing enhanced inhibitory activity in vitro [80,189]. These structural modifications aim to balance potency, selectivity, and safety, although advanced clinical evaluation still remains to be proven.

**Table 2:** Comparative Overview of GABA-T Inhibitors.

Inhibitor	Mechanism of Action	Reversibility	Key Features	Clinical/Development Status	References
Vigabatrin (γ-vinyl-GABA)	Mechanism-based irreversible inhibition (suicide substrate forming covalent adduct with PLP)	Irreversible	Potent CNS penetration; elevates GABA levels 100–200%; risk of retinal toxicity	FDA-approved for infantile spasms and refractory partial seizures	[190,191]
Cyclic GABA analogs	Conformationally restricted inhibitors	Reversible	Modest activity; low potency	Experimental	[112,192,193]
OV101 (Gaboxadol)	Selective agonist at extrasynaptic GABA_A_ receptors containing the δ-subunit; enhances tonic inhibition without directly affecting synaptic (phasic) transmission.	Reversible	Acts independently of GABA-T inhibition; increases inhibitory tone through sustained activation of high-affinity GABA_A_ receptors; provides a novel approach to restoring inhibitory balance.	Originally developed for insomnia; later investigated for epilepsy, Angelman syndrome, and Fragile X syndrome. Phase II clinical trials showed mixed efficacy; development for epilepsy was discontinued, but research in neurodevelopmental disorders continues.	[194,195,196]
CPP-115	Vigabatrin analog with improved CNS selectivity and reduced peripheral exposure	Irreversible	Designed to lower retinal toxicity; greater potency at lower doses	Early clinical stage	[197,198,199]
Ethanolamine O-sulfate	GABA analog; competitive inhibition of GABA-T	Reversible	Mild anticonvulsant effect; limited potency	Preclinical	[200,201,80]
γ-Acetylenic GABA	Mechanism-based inactivator similar to vigabatrin	Irreversible	High in vitro potency; safety not established	Experimental	[202,203]
Fluorinated GABA analogs	Modified lipophilicity and enzyme affinity	Reversible or irreversible (depending on analog)	Potentially enhanced selectivity	Preclinical	[204,205,206]

### 6.3. Comparative Pharmacology of Reversible and Irreversible Inhibition

Irreversible GABA-T inhibitors such as vigabatrin provide sustained enzyme inactivation, producing long-lasting effects even after treatment cessation. This confers dosing convenience and robust efficacy but also prolongs adverse outcomes, particularly visual toxicity [32,112]. In contrast, reversible inhibitors allow finer dose control and rapid offset of action, theoretically improving safety. However, achieving durable seizure suppression with reversible inhibition remains difficult because of limited residence time and rapid GABA turnover [112,207]. To date, reversible inhibitors have not demonstrated clinical superiority over vigabatrin; however, they may offer an improved side effect profile, particularly with potentially reduced risk of irreversible visual toxicity, though clinical data remain limited.

### 6.4. Structure–Activity Relationships of GABA-T Inhibitors

Medicinal chemistry efforts have revealed key structural determinants governing GABA-T inhibition. The amino acid backbone, containing both amino and carboxyl groups in specific spatial orientation, is essential for enzyme recognition. GABA’s four-carbon chain provides an optimal fit within the active site, whereas shorter or longer analogs show reduced activity. Substituents at the γ-position, such as the vinyl group in vigabatrin, act as reactive centers enabling mechanism-based inactivation, and variations in these groups markedly influence potency.

Stereochemistry plays a critical role, as only the S(+) enantiomer of vigabatrin exhibits significant activity, approximately 200-fold higher than the R(–) form, highlighting the enzyme’s strict stereospecificity. Lipophilicity determines CNS penetration but must be balanced against solubility and peripheral distribution. Incorporation of heterocycles or ring systems has been employed to optimize binding affinity and pharmacokinetics, provided that the essential pharmacophoric elements are retained [208].

### 6.5. Experimental and Dual-Action GABA-T Inhibitors

Several experimental compounds have demonstrated promising GABA-T inhibitory activity in preclinical studies. Ethanolamine O-sulfate shows moderate inhibition and anticonvulsant effects but lacks sufficient potency for clinical utility [209,210]. γ-Acetylenic GABA derivatives act via mechanism-based enzyme inactivation similar to vigabatrin, with some analogs displaying superior in vitro potency; however, their in vivo safety remains uncertain [211]. Cyclic GABA analogs designed to restrict conformational flexibility have shown reversible inhibition but modest activity overall [212].

Dual-action molecules that combine GABA-T inhibition with other mechanisms, such as GAT-1 inhibition or GABA_A_ receptor modulation, are also under exploration [68]. These agents aim to enhance efficacy through synergistic effects but introduce additional challenges in selectivity and safety assessment.

### 6.6. Outlook and Challenges in GABA-T Inhibitor Development

Developing next-generation GABA-T inhibitors require demonstrating clear clinical advantages over vigabatrin, a difficult benchmark given its established efficacy in select epilepsies. New agents must achieve comparable seizure control while markedly reducing retinal toxicity, a task requiring extensive preclinical safety validation. Moreover, the relatively small patient population for conditions such as infantile spasms limit commercial incentives. Nonetheless, advances in structural biology, computational design, and targeted delivery systems offer promising avenues for generating safer and more effective GABA-T inhibitors.

## 7. Emerging Research and Future Directions

### 7.1. Innovation in Inhibitor Design and Discovery Strategies

The therapeutic success of vigabatrin has spurred ongoing interest in developing next-generation GABA-T inhibitors with improved safety and selectivity. Advances in structural biology and computational chemistry have enabled more precise structure-based drug design, helping to elucidate key molecular interactions required for selective inhibition [213,214]. Current efforts focus on optimizing CNS specificity while reducing retinal exposure by modulating lipophilicity, utilizing CNS-specific prodrug strategies, and leveraging selective transporters at the blood–brain barrier. Additionally, reversible inhibitors with tunable kinetics are being explored to maintain therapeutic efficacy while allowing faster enzyme recovery upon treatment withdrawal, thereby mitigating cumulative toxicity [215,216,217].

Complementary to these developments, computer-aided drug discovery methods, particularly molecular docking, virtual screening, and related simulation-based approaches, have broadly facilitated the identification and optimization of novel molecular scaffolds across diverse therapeutic targets [218,219,220,221,222,223,224,225], supporting the discovery of candidates with improved pharmacokinetic and pharmacodynamic potential, including emerging compounds designed to modulate GABA metabolism and selectively inhibit GABA-T [213,226,227,228,229]. Fragment-based drug discovery has further expanded chemical diversity and enabled the rational assembly of small fragments into more potent and selective inhibitors. Together, these integrative discovery approaches provide promising starting points for designing safer and more effective GABA-T-targeted therapies [230,231].

### 7.2. Targeted Delivery and Multi-Mechanistic Therapeutic Strategies

Efforts to improve the clinical safety of GABA-T inhibitors have also turned toward advanced drug delivery and therapeutic combination strategies. Innovative nanocarrier systems aim to enhance brain selectivity while reducing peripheral accumulation, particularly in ocular tissues implicated in vigabatrin-associated retinal toxicity [232]. Localized CNS delivery methods, though largely preclinical, are being explored to restrict drug exposure to epileptogenic brain regions and minimize systemic adverse effects [233,234].

At the same time, rational polytherapy is gaining interest as a strategy to enhance efficacy while lowering individual drug burden. Combining GABA-T inhibitors with agents targeting complementary pathways, such as GABA_A_ receptors, glutamatergic signaling, or neuroprotective mechanisms, may produce synergistic effects at reduced doses. Multi-target drug designs that integrate transporter modulation or receptor interactions with GABA-T inhibition also show promise for achieving more balanced neurochemical regulation. Adjunctive use of antioxidants, mitochondrial stabilizers, or taurine has been proposed as a means to mitigate retinal toxicity associated with long-term vigabatrin therapy [235,236,237].

### 7.3. Biomarkers, Neuroprotection, and Expanding Clinical Applications

A major future direction in the field involves identifying reliable biomarkers to guide individualized therapy and predict treatment response. Neuroimaging tools such as magnetic resonance spectroscopy can quantify brain GABA levels, whereas functional MRI and PET imaging offer complementary insights into GABAergic network activity and receptor occupancy [238,239]. EEG-based biomarkers, particularly gamma oscillations and event-related potentials, are being investigated for early prediction of therapeutic response in conditions such as infantile spasms. Molecular and genetic markers reflecting GABA metabolism, neuroinflammation, or retinal vulnerability may ultimately support more personalized treatment strategies [240,241].

Beyond seizure suppression, emerging evidence suggests that GABA-T inhibition may exert neuroprotective or anti-epileptogenic effects. Preclinical models show that early intervention can modulate neuroinflammation, reduce aberrant synaptic remodeling, and attenuate neuronal injury, raising the possibility of delaying or preventing chronic epilepsy development. Clinically, early vigabatrin use—especially in infants with tuberous sclerosis complex—has been associated with improved developmental outcomes, although the degree to which this reflects true anti-epileptogenic activity remains under investigation [242,243,244].

Finally, modulation of GABA metabolism is under exploration for therapeutic applications beyond epilepsy. Preliminary studies have assessed vigabatrin and related compounds in substance-use disorders, particularly cocaine and methamphetamine dependence, where enhancing GABAergic inhibition may dampen dopaminergic reward pathways. Additional early investigations have considered potential benefits in neuropathic pain, movement disorders, anxiety, and depression. Continued development of safer, reversible, and more targeted GABA-T inhibitors could revive and expand research into these alternative clinical indications [245,246].

## 8. Challenges and Limitations

Current monitoring of vigabatrin therapy primarily relies on visual field testing, which can detect retinal damage only after it has occurred and depends heavily on patient cooperation. This makes it particularly unreliable for children, infants, and individuals with cognitive impairment. The test is also subject to variability, learning effects, and significant resource and time demands, limiting its practicality for preventive screening. In pediatric cases, especially in infantile spasms, the urgency of rapid seizure control must be balanced against the risk of potentially irreversible visual toxicity, with treatment decisions often relying on caregiver consent because early ophthalmologic assessment is difficult.

A key challenge arises from the mechanism underlying vigabatrin-associated visual toxicity. Vigabatrin irreversibly inhibits GABA transaminase (GABA-T), increasing GABA concentrations systemically, including in the retina. Excessive retinal GABA may disrupt normal neurotransmission, alter photoreceptor function, and contribute to mitochondrial stress and oxidative damage in retinal cells. Additionally, vigabatrin has been shown to accumulate in the retina at higher levels than in the brain, possibly due to distinct transporter activity, further amplifying toxicity risk. Because the drug binds irreversibly, both therapeutic and adverse effects persist long after dosing, complicating adjustments and making washout studies difficult.

Interindividual responses to vigabatrin vary widely, and no reliable biomarkers exist to predict patients who are most susceptible to visual field loss. Long-term data on visual, cognitive, and developmental outcomes remain limited, highlighting the need for extended and systematic follow-up. Required ophthalmologic monitoring increases the cost and reduces accessibility of vigabatrin therapy, particularly in low-resource settings.

These limitations emphasize the urgent need for safer, more selective GABA-T inhibitors. Novel compounds with improved tissue selectivity, reduced retinal accumulation, reversible binding mechanisms, or lower propensity to elevate retinal GABA levels may retain therapeutic efficacy while significantly reducing toxicity risk. Advances in rational drug design, retinal-specific pharmacokinetic modeling, and biomarker discovery may help guide the development of next-generation GABA-T-targeted therapies that overcome the major barriers associated with vigabatrin.

## 9. Conclusions

GABA aminotransferase remains a well-validated and clinically important therapeutic target in epilepsy, as demonstrated by the success of vigabatrin in treating infantile spasms and refractory partial seizures. Its role in GABA catabolism makes it ideal for enhancing inhibitory neurotransmission, and vigabatrin’s mechanism-based irreversible inhibition provides sustained seizure control through long-lasting elevation of brain GABA levels. However, this same property underlies its major limitation, the risk of irreversible visual field defects, necessitating cautious and selective clinical use. Despite these drawbacks, the overall therapeutic principle of GABA-T inhibition remains robust. Vigabatrin’s clinical impact highlights the potential of mechanism-driven drug design, while also underscoring the critical need for improved safety and individualized treatment strategies. Future research is focused on developing reversible or more selective GABA-T inhibitors that maintain efficacy but reduce retinal toxicity, as well as innovative delivery methods to enhance CNS targeting while minimizing peripheral exposure. Furthermore, integrating pharmacogenomic and biomarker-based approaches may help identify patients most likely to benefit with minimal risk, paving the way for precision medicine in epilepsy care. The ongoing exploration of combination therapies and neuroprotective adjuncts also offers opportunities to optimize outcomes and mitigate adverse effects.

Ultimately, the legacy of vigabatrin extends beyond its own clinical utility, it exemplifies how a deep understanding of neurochemical pathways can yield transformative therapies while revealing the complexities of balancing efficacy and safety. Continued research into GABA metabolism, enzyme regulation, and patient-specific factors will likely sustain GABA-T inhibition as a cornerstone concept in antiepileptic drug development, guiding the creation of next-generation treatments that offer the benefits of enhanced GABAergic function with improved safety and tolerability.

## Data Availability

No new data were created or analyzed in this study. Data sharing is not applicable to this article.
